# Characteristics of the Urinary Microbiome From Patients With Gout: A Prospective Study

**DOI:** 10.3389/fendo.2020.00272

**Published:** 2020-05-20

**Authors:** Yaogui Ning, Guomei Yang, Yangchun Chen, Xue Zhao, Hongyan Qian, Yuan Liu, Shiju Chen, Guixiu Shi

**Affiliations:** ^1^School of Medicine, Xiamen University, Xiamen, China; ^2^Department of Intensive Care Unit, The First Affiliated Hospital of Xiamen University, Xiamen, China; ^3^Department of Rheumatology and Clinical Immunology, The First Affiliated Hospital of Xiamen University, Xiamen, China

**Keywords:** gout, urine, microbiome, 16S rRNA, high-throughput sequencing, biomarker

## Abstract

The role of host microbes in the pathogenesis of several diseases has been established, and altered microbiomes have been related to diseases. However, the variability of the urinary microbiome in individuals with gout has not been evaluated to date. Therefore, we conducted the present prospective study to characterize the urinary microbiome and its potential relation to gout. Urine samples from 30 patients with gout and 30 healthy controls were analyzed by Illumina MiSeq sequencing of the 16S rRNA hypervariable regions, and the microbiomes were compared according to alpha-diversity indices, complexity (beta diversity) with principal component analysis, and composition with linear discriminant analysis effect size. The most significantly different taxa at the phylum and genus levels were identified, and their potential as biomarkers for discriminating gout patients was assessed based on receiver operating characteristic (ROC) curve analysis. Compared with the healthy controls, there was a dramatic decrease in microbial richness and diversity in the urine of gout patients. The phylum Firmicutes and its derivatives (Lactobacillus_iners, Family_XI, and Finegoldia), the phylum Actinobacteria and its derivatives (unidentified_Actinobacteria, Corynebacteriales, Corynebacteriale, Corynebacterium_1, and Corynebacterium_tuberculostearicum), and the genera *Prevotella* and Corynebacterium_1 were significantly enriched in the urine of gout patients. ROC analysis indicated that the top five altered microbial genera could be reliable markers for distinguishing gout patients from healthy individuals. These findings demonstrate that there are specific alterations in the microbial diversity of gout patients. Thus, further studies on the causal relationship between gout and the urinary microbiome will offer new prospects for diagnosing, preventing, and treating gout.

## Introduction

The human microbiome is increasingly considered an important contributor to both health and diseases (1, 2). Advanced technologies for the sequencing of bacterial 16S rRNA alleles have revealed the existence of culture-independent microbes in areas of the human body previously considered to be microbe-free, including urine (3–6). Indeed, several studies have demonstrated a diverse microbiome in the human mouth, skin, respiratory system, gastrointestinal tract, and urine, including previously uncharacterized and uncultivated species (7, 8). Moreover, accumulating evidence since the completion of the National Institutes of Health Human Microbiome Project points to an association between dysbiosis of the microbiome and the pathogenesis of multiple diseases, including gastrointestinal diseases, pulmonary diseases, cancer, metabolic diseases, and inflammatory diseases (1, 9, 10). Thus, further comprehensive study of the microbiome characteristics related to diseases can provide new insights into the pathogenic mechanism or inform new strategies for diagnosis and treatment.

Gout is one of the most common auto-inflammatory diseases, which is characterized by elevated levels of serum uric acid (UA) with consequent deposition of urate in and around the joints. The prevalence of gout is around 3.9% in the USA and 2.5% in Europe (11–13). Although the specific pathogenic mechanism of gout remains unclear, disruption of purine metabolism and inflammation regulation has been implicated. Treatment options are also limited, and patients with gout require the use of long-term drugs to decrease the UA level. UA is excreted through the kidneys and intestine, and several studies have indicated the abnormal excretion of UA in gout, which could potentially alter the microbiome. Moreover, the microbiome itself might contribute to the abnormal UA metabolism or inflammation regulation in gout. Thus, understanding the characteristics of the microbiome in gout patients might provide potential new strategies for diagnosis and help elucidate the pathogenic mechanisms.

A previous study demonstrated the presence of intestinal bacterial dysbiosis in gout patients compared with a healthy population (14). However, the characteristics of the microbiome in the urine of gout patients remain unclear. In this study, Illumina MiSeq sequencing was employed to investigate the microbial community in urinary extracts from gout patients and healthy volunteers to explore the urinary microbiome alterations associated with gout. These results can serve as a resource for further research to gain a better understanding of the role of bacterial dysbiosis in the pathogenic mechanism and identify candidate diagnostic biomarkers for gout.

## Materials and Methods

### Study Design and Subject Recruitment

The study cohort consisted of two groups (Table 1): the gout group (*n* = 30) and the healthy group (*n* = 30). Gout was diagnosed by rheumatologists according to the American College of Rheumatology (ACR)/EULAR 2015 criteria (15). The patients and healthy volunteers were (all males) were recruited from the First Affiliated Hospital of Xiamen University, China, from March to October 2017. The exclusion criteria were follows: (1) any comorbid disorders and (2) taking antibiotics within 1 month prior to enrolment in the study. The study was approved by the Ethics Committee of the First Affiliated Hospital of Xiamen University, and informed consent was obtained from all participants. Demographic characteristics, including age, body mass index, smoking, alcohol intake, or dietary habits, and laboratory data were recorded for all subjects.

**Table 1:** Characteristics of gout patients and healthy controls.

**Characteristic**	**Gout patients (*n* = 30)**	**Healthy controls (*n* = 30)**	***P*-value**
Age	45.86 ± 9.84	41.36 ± 14.30	0.161
BMI (kg/m^2^)	24.09 ± 1.41	23.12 ± 1.30	0.203
UA (μmol/L)	456.30 ± 72.94	265.73 ± 68.19	0.038
BUN (mmol/L)	6.62 ± 1.40	4.43 ± 1.00	0.000
Cr (μmol/L)	73.00 ± 15.08	69.23 ± 16.97	0.426
Average reads	79,785.03 ± 3982.98	78,727.63 ± 5892.81	0.430

*Values are presented as mean ± SD. BMI, body mass index; UA, ureic acid; BUN, urea nitrogen; Cr, creatinine*.

### Sample Preparation and DNA Extraction

Mid-stream urine samples freshly collected from each individual were immediately frozen at −20°C and transported to the laboratory with an ice pack. Total bacterial DNA from samples was extracted at Novogene Bioinformatics Technology Co., Ltd. using a TIANGEN kit according to the manufacturer's protocols. The quality of the extracted DNA was determined by 1% agarose gel electrophoresis, and the optical density value at 260/280 nm was measured on a spectrophotometer. According to the concentration, the DNA was diluted to 1 ng/μL using sterile water and stored at −20°C for Illumina MiSeq sequencing analysis.

### Polymerase Chain Reaction (PCR) Amplification of the Bacterial 16S rRNA V3–V4 Region and Illumina Pyrosequencing

The V3–V4 hypervariable region of the 16S rRNA gene was amplified from the diluted DNA extracts with the forward primer 341F and reverse primer 806R. All PCRs were carried out in 30 μL reactions with 15 μL of Phusion® High-Fidelity PCR Master Mix (New England Biolabs), 0.2 μM of forward and reverse primers, and about 10 ng of template DNA. The thermal cycling program consisted of initial denaturation at 98°C for 1 min, followed by 30 cycles of denaturation at 98°C for 10 s, annealing at 50°C for 30 s, and elongation at 72°C for 30 s, followed by a final extension step at 72°C for 5 min. The same volume of 1X loading buffer (containing SYBR Green) was mixed with the PCR products and subjected to electrophoresis on a 2% agarose gel for detection. Samples with a bright main strip between 400 and 450 bp were chosen for further analysis.

The PCR products were mixed at equidensity ratios and purified with GeneJET Gel Extraction Kit (Thermo Scientific). Sequencing libraries were generated using TruSeq® DNA PCR-Free Sample Preparation Kit according to the manufacturer's recommendations, and index codes were added. The library quality was assessed on a Qubit® 2.0 fluorometer (Thermo Scientific) and an Agilent Bioanalyzer 2100 system. Finally, the library was sequenced on an Illumina HiSeq 2500 system, and 250-bp paired-end reads were generated.

### Statistics

#### Clinical Data Analysis

Quantitative demographic data with a normal distribution are expressed as mean ± standard deviation, and the *t*-test was used for comparisons between the two groups. All statistical tests were two-sided, and *P* <0.05 was regarded as statistically significant. Statistical analyses were performed using SPSS 19 (SPSS, Chicago, IL, USA).

#### Bioinformatics Analysis

Alpha diversity was used to analyze the complexity of species diversity for a sample through six indices: observed-species, Chao1, Shannon, Simpson, ACE, and Good's coverage. All indices in our samples were calculated with QIIME (version 1.7.0), and the results were displayed with the R software (version 2.15.3). Chao1 and observed-species represent bacterial richness, whereas the Shannon and Simpson indices are quantitative measures of bacterial diversity reflecting both species richness and evenness. The difference in alpha diversity was evaluated by the Kruskal–Wallis test.

Beta diversity was used to evaluate differences in species complexity among samples. Beta diversity on both weighted and unweighted UniFrac distances was calculated by the QIIME software (version 1.7.0). Principal coordinates analysis (PCoA) was performed to obtain principal coordinates and reduce the complex, multidimensional data for visualization of patterns and to assess whether urinary microbial species could be differentiated between gout patients and healthy controls. A distance matrix of weighted or unweighted UniFrac distances among samples was transformed to a new set of orthogonal axes, by which the maximum variation factor is reflected by the first principal coordinate, the second maximum factor is reflected by the second principal coordinate, and so on. PCoA results were displayed using the WGCNA package, stat packages, and ggplot2 package in the R software (version 2.15.3).

Each sample was mapped based on the overall microbial composition and assessed for similarities. To identify significantly different bacteria as biomarkers between groups, the online software linear discriminant analysis effect size (LEfSe) (16) was utilized to select and demonstrate differentially abundant taxonomic groups based on the Kruskal–Wallis test and linear discriminant analysis (LDA) score.

Moreover, receiver operating characteristic (ROC) curves were constructed using the statistical programming language R (V.3.1.2) to visualize the potential of significantly altered genera as gout biomarkers. ROC curves are used to evaluate the performance or the quality of diagnostic tests and are widely used to evaluate the performance of many microbial biomarkers in gut microbiome analyses (17, 18). Area under the curves (AUCs) of ROC was generated to evaluate the performance of the fitted logistic regression models. It was based on the predicted probability of gout for each individual by the multivariate logistic regression coefficient estimates and the individual's transformed relative abundances for each bacterial taxon included in the analysis to predict the probability of gout for each individual.

## Results

### Subject Characteristics

The basic characteristics of the patients (*n* = 30) and healthy controls (*n* = 30) are summarized in Table 1. All of the subjects were male, with no significant differences between the two groups in terms of age, smoking history, alcohol intake, or dietary patterns. No significant difference between the groups was found in the laboratory data except for the serum levels of UA and blood urea nitrogen, which were both significantly elevated in the gout group.

### Urinary Dysbiosis in Gout Patients

The urine samples of all 60 subjects were analyzed to assess overall differences in the microbial community structure in urine between gout patients and healthy individuals. After optimization, a total of 4,755,380 sequence reads were included in the final analysis. The results of Illumina MiSeq sequencing showed at least 64,967 valid reads of each sample for operational taxonomic unit (OTU) analysis.

**Figure 1:**
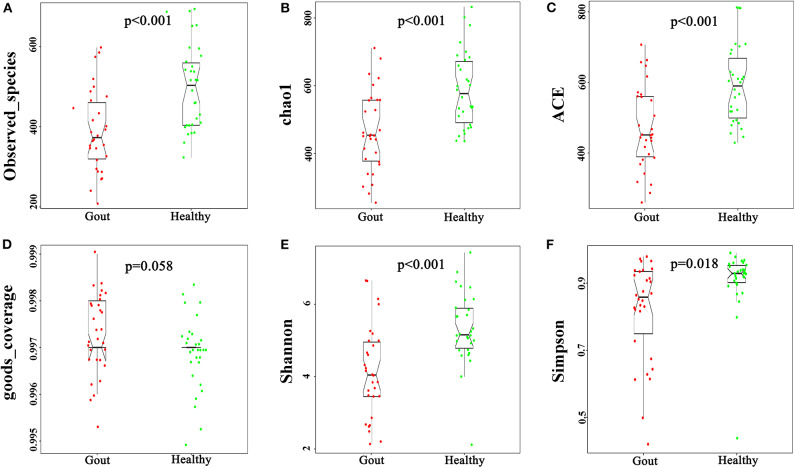
Variation in alpha-diversity indices between gout patients (red) and healthy controls (green). Six indices of alpha-diversity were analyzed. The results demonstrate that ACE, Chao1, Shannon, Simpson, and observed-species of the urinary microbiota from the gout group were significantly lower than those of the healthy group **(A–C,E,F)**, while no statistical difference in Good's coverage was found **(D)**.

The urinary microbiota of gout patients was significantly different from that of healthy controls. Most of the alpha-diversity indices (ACE, Chao1, Shannon, Simpson, and observed-species) of the urinary microbiota from the gout group were significantly lower than those of the healthy group (Figure 1). Good's coverage was higher in the gout patients than in the healthy controls, but there was no statistical difference (*P* = 0.058). These results indicate that gout patients have a lower diversity and richness in the urinary microbiome, but the evenness of the urinary microbiome is similar to that of healthy controls.

**Figure 2:**
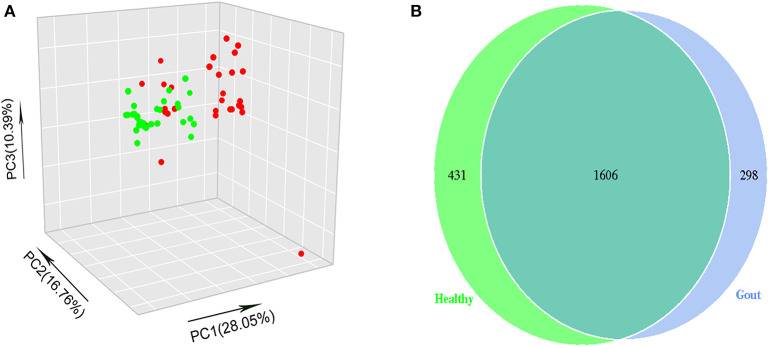
**(A)** The beta-diversity indices between gout patients (red) and healthy controls (green) are shown in PCoA analysis. The ordination plot shows a clear difference between the two groups. **(B)** Venn diagrams show the percentage of shared OTUs between gout patients and healthy controls. The results show that a total of 1606 OTUs were shared between two groups, which accounted for 84.34% of the total OTUs in healthy controls and 78.84% of the total OTUs in gout patients.

The difference in beta diversity according to the weighted UniFrac distance was assessed by PCoA, which indicated that most of the samples from the two groups clustered together (Figure 2A). However, the ordination plot demonstrates difference between the gout patients and healthy controls. Further examination showed that a total of 1606 OTUs were shared between the gout patients and healthy controls, which accounted for 84.34 and 78.84% of the total OTUs in healthy controls and gout patients, respectively (Figure 2B).

### Major Differences Between the Microbiomes of Healthy Individuals and Gout Patients

**Figure 3:**
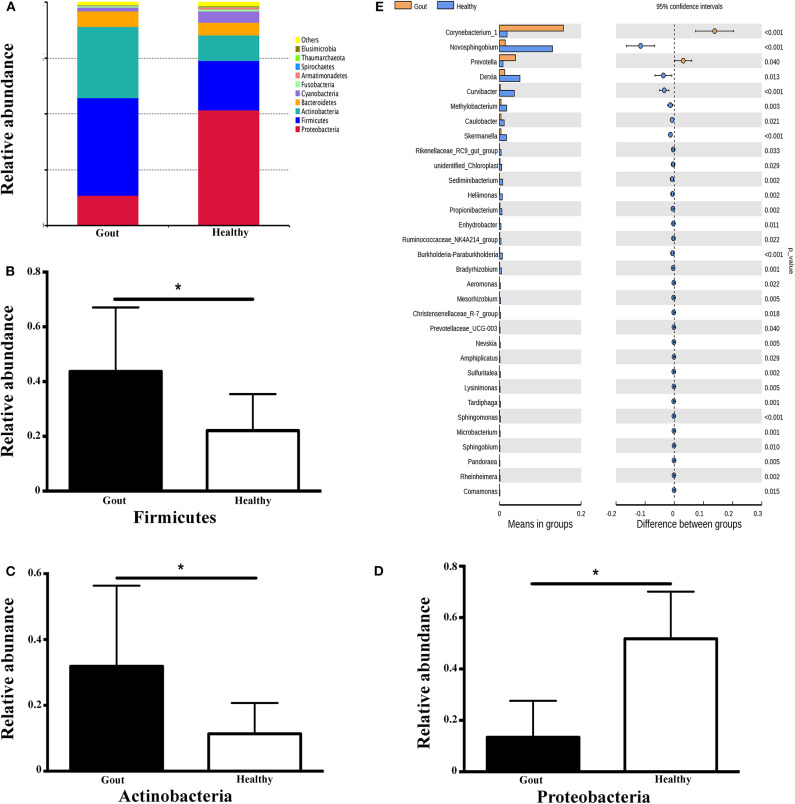
Microbiota composition at the phylum and genus levels. **(A)** Relative abundances of the 10 dominant bacterial phyla found across the two groups shown as histograms. A *t*-test was used to detect the difference between the two groups. **(B–D)** Phyla with significantly different relative abundances between two groups, *P <0.05. **(E)** Genus with significantly different relative abundances between two groups. Different genera were assigned only to those presenting minimum variation at a significance level of *P* <0.05.

The range of sequence reads obtained per subject varied from 64,967 to 90,070. A total of 30,488 OTUs were observed across all subjects, and the range of OTU numbers varied from 255 to 769. A total of 10 bacterial phyla accounted for >90% of all sequence reads in the two groups (Figure 3A). At the phylum level, the relative abundances of Firmicutes and Actinobacteria in the gout group were significantly higher than those in the controls (*P* <0.001) (Figures 3B,C). However, the other dominant phyla showed lower abundances in the gout group, especially the relative abundance of Proteobacteria (Figure 3D).

At the genus level, Corynebacterium_1 was clearly enriched in the gout patients (*P* = 0.004), which possibly inflated the total Actinobacteria abundance observed at the phylum level. Another minor genus that was also significantly more abundant in the gout patients was *Prevotella*, which belongs to the phylum Bacteroidetes (*P* = 0.04). The relative abundances of other genera, including *Novosphingobium, Derxia, Curvibacter, Methylobacterium, Caulobacter, Skermanella*, unidentified_Chloroplast, and Rikenellaceae_RC9_gut_group, were significantly decreased in gout patients (Figure 3E).

### Potential Biomarkers to Differentiate Gout Patients From Healthy Patients

**Figure 4:**
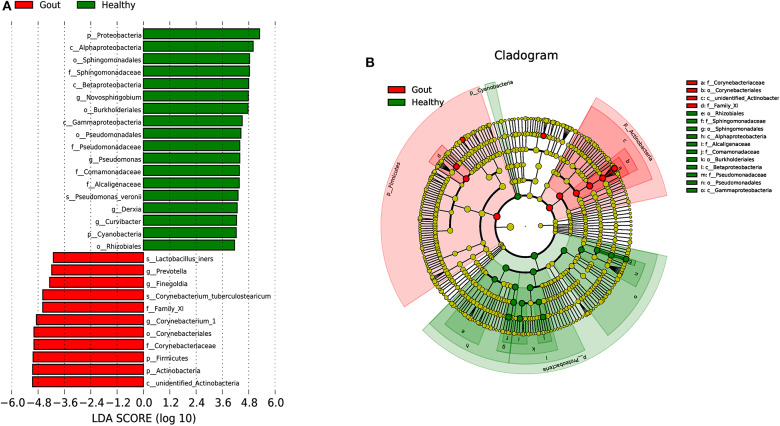
A cladogram representative of the structure of the urinary microbiota and the predominant bacteria. **(A)** Histograms of LDA score; red and green represent gout samples and healthy controls, respectively. **(B)** Cladogram showing differentially abundant taxa of urinary microbiota in gout patients and healthy controls.

To explore the potential gout-associated biomarkers, the urinary microbiome sequence data were subjected to LEfSe analysis. A cladogram representative of the structure of the urinary microbiota and the predominant bacteria is shown in Figure 4, which also displays the taxa with the greatest differences between the two groups and the discrepant microbial species with a reduced significance threshold (LDA score > 2). The LEfSe method revealed that the phylum Firmicutes and its derivatives (Lactobacillus_iners, Family_XI, and Finegoldia), the phylum Actinobacteria and its derivatives (unidentified_Actinobacteria, Corynebacteriales, Corynebacteriale, Corynebacterium_1, and Corynebacterium_tuberculostearicum), and the genus *Prevotella* all showed higher relative abundances in the urinary microbiota from the gout patients, suggesting these taxa as candidate biomarkers for potential distinguishing between gout patients and healthy controls.

**Figure 5:**
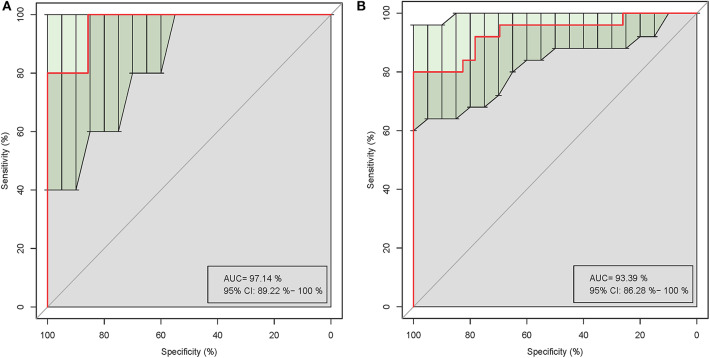
Receiver operating characteristic (ROC) curves demonstrating the performance of significantly altered microbial genera for the test **(A)** and training **(B)** validation subjects using leave-one-out cross-validation.

Five of the significantly altered genera (*Pandoraea, Curvibacter, Skermanella, Novosphingobium*, and *Sulfuritalea*) were screened for their abilities in distinguishing gout patients and healthy controls in the ROC analysis. Complete results of the training and test validation subjects using leave-one-out cross-validation are shown in Figure 5. This combination of significantly altered genera could effectively differentiate between gout patients and healthy controls with AUC values of 0.971 [95% confidence interval (CI) 89.22–100%] in the test validation subjects (Figure 5A) and 0.934 (95% CI 86.28–100%) in the training validation subjects (Figure 5B).

## Discussion

In this prospective pilot study, we first characterized the urinary microbiome of male patients with gout and healthy individuals using high-throughput sequencing of the V3–V4 region of the 16S rRNA gene. The results showed clear differences in the microbiomes between the groups, with lower diversity and richness in the gout patients, which might contribute to the abnormal UA metabolism and inflammation regulation in gout. The combination of significantly altered genera showed good ability to differentiate gout patients from healthy controls, which might provide a new non-invasive biomarker for improving gout diagnosis.

Our results further highlight the polymicrobial composition of human urine, with evident individual variation. The OTU analysis suggested that the microbiota of gout samples showed over 79% similarity with that of healthy controls. The alpha-diversity (observed-species, Chao1, ACE, Shannon, and Simpson) indices of the urinary microbiota from the gout group were all lower than those of the healthy controls, suggesting a decrease in the overall richness and ecological diversity in gout patients. These slight differences spanned from the phylum level to the species level. All of the urine samples were predominantly composed of bacteria from four phyla, Firmicutes, Proteobacteria, Actinobacteria, and Bacteroidetes, which is consistent with the findings of Karstens et al. (19). The most abundant phylum in healthy controls was Proteobacteria, whereas Firmicutes was the most abundant phylum in the gout group. Firmicutes is considered as the most abundant bacteria in normal urine samples (19–23), although Proteobacteria has also been reported to be the most common in healthy urine (24). This overall similarity at the phylum level could suggest a relatively stable bacterial community in gout. However, at the genus level, more specific and clear shifts were observed, especially for genera in Actinobacteria and Bacteroidetes. The predominant ecological structure of the urine microbiomes showed slight variations in relative abundance in the two groups. In particular, *Prevotella* increased in the gout patients, which belongs to Bacteroidetes, and was reported to be the second and/or third most abundant or even lower in rank in healthy individuals (21, 22, 25). A previous study also demonstrated that the family Bacteroidaceae and its genus *Bacteroides* were enriched in the gut of male gout patients (26). *Prevotella* has been regarded as pathogenic in the vaginal microbiome (27). This genus was also found to be was enriched in patients with type 2 diabetes (24) and in general non-healthy individuals (21, 25). *Prevotella* was also found to be increased in the gut microbiome of patients with kidney stones compared with non-stone controls (28). Bacteria selectively aggregate to crystals in the urinary tract, which suggests a proper mechanistic role for bacteria in stone formation. Gout is a high-risk factor for the formation of kidney stones; however, the role of *Prevotella* in gout and/or uric acid stone formation remains unknown.

Corynebacterium_1 was also a predominant genus detected in the gout group, whereas *Novosphingobium* was more predominant in the urine of healthy controls. The abundance of Corynebacterium_1 was previously demonstrated to be correlated with serum concentrations of interleukin-6 and C-reactive protein in cancer patients (29). Gout not only is a metabolic condition but is also an auto-inflammatory disease associated with an increased inflammatory reaction. Therefore, these findings could suggest an association between urinary microbiota such as Corynebacterium_1 and an inflammatory reaction. The relative abundance of Corynebacterium_1 was previously reported to be increased in patients with prostate cancer (30). Both gout and prostate cancer are clinical conditions that mainly occur in male patients; therefore, it would be interesting to investigate a potential sex-specific pattern of Corynebacterium_1 abundance. The urine samples of the present study were all from male subjects since gout is predominant in men. However, previous studies have demonstrated a difference in the healthy urine microbiome of males and females (31). Therefore, these conclusions should be interpreted with caution when applied to elderly female patients suffering from gout.

There is previous evidence of an association of the gut microbiota with gout (14, 26, 32), suggesting that the altered metabolites of gout patients may play a role not only in inflammation disorders but also in purine metabolism and UA excretion. The intestinal microbiota composition also markedly varies according to dietary intake (33), which could induce differences in both the microbiota composition and microbial metabolites (34). Consequently, the urinary microbiota can be indirectly influenced by gut microbiota alterations caused by dietary patterns (35).

Here, we found some specific alterations of the urinary microbiome with potential to distinguish gout patients from healthy controls. In particular, the combination of five genera that were significantly altered in the urine of gout patients, *Pandoraea, Curvibacter, Skermanella, Novosphingobium*, and *Sulfuritalea*, could effectively distinguish gout patients from healthy controls with a high predictive value. To the best of our knowledge, this is the first attempt to characterize the urinary signatures of gout by integrating the microbiome. The significantly altered urine microbiome could serve as a biomarker to discriminate between gout patients and healthy controls, thereby improving diagnosis and allowing for early intervention. Further exploration of the underlying mechanisms to explain these associations could also provide insight into the pathogenesis of gout and suggest new treatment strategies.

## Data Availability Statement

Raw Illumina data reads are available in the NCBI Sequence Read Archive database of GenBank under accession ID SRP153570.

## Ethics Statement

The studies involving human participants were reviewed and approved by the Ethics Committee of the First Affiliated Hospital of Xiamen University. The patients/participants provided their written informed consent to participate in this study. Written informed consent was obtained from the individual(s) for the publication of any potentially identifiable images or data included in this article.

## Author Contributions

GS participated in the research design and data analysis, reviewed and revised the manuscript, and provided general supervision and financial support. YL revised the manuscript. YN performed data acquisition, sample collection and analysis, and interpretation and contributed to article drafting and revision. SC performed sample collection, data analysis, and article revision and provided financial support. GY, XZ, HQ, and YC performed data acquisition and sample collection. All authors contributed to the study and approved the final submitted manuscript.

## Conflict of Interest

The authors declare that the research was conducted in the absence of any commercial or financial relationships that could be construed as a potential conflict of interest.

## Abbreviations

UA: ureic acid
ROC: receiver operating characteristic
LDA: linear discriminant analysis
LEfSe: linear discriminant analysis effect size
PCoA: principal coordinates analysis
AUC: area under the curve.
